# Development of a semi-customized tongue displacement device using a 3D printer for head and neck IMRT

**DOI:** 10.1186/s13014-019-1289-x

**Published:** 2019-05-14

**Authors:** Chae-Seon Hong, Dongryul Oh, Sang Gyu Ju, Yong Chan Ahn, Cho Hee Na, Dong Yeol Kwon, Cheol Chong Kim

**Affiliations:** 1 Department of Radiation Oncology, Samsung Medical Center, Sungkyunkwan University School of Medicine, Irwon-Ro 81 ,Gangnam-Gu, Seoul, 06351 Republic of Korea; 2 Department of Radiation Oncology, Yonsei Cancer Center, Yonsei University College of Medicine, Seoul, Republic of Korea; 3 Department of Medical Device Management and Research, Samsung Advanced Institute for Health Science & Technology, Sungkyunkwan University, Seoul, Republic of Korea

**Keywords:** Tongue displacement, 3D printing, Head and neck cancer, Tomotherapy

## Abstract

**Purpose:**

To reduce radiation doses to the tongue, a patient-specific semi-customized tongue displacement device (SCTDD) was developed using a 3D printer (3DP) for head and neck (H&N) radiation therapy (RT). Dosimetric characteristics of the SCTDD were compared with those of a standard mouthpiece (SMP).

**Materials and methods:**

The SCTDD consists of three parts: a mouthpiece, connector with an immobilization mask, and tongue displacer, which can displace the tongue to the contralateral side of the planning target volume. Semi-customization was enabled by changing the thickness and length of the SCTDD. The instrument was printed using a 3DP with a biocompatible material. With the SCTDD and SMP, two sets of planning computed tomography (CT) and tomotherapy plans were obtained for seven H&N cancer patients. Dosimetric and geometric characteristics were compared.

**Results:**

Using the SCTDD, the tongue was effectively displaced from the planning target volume without significant tongue volume change compared to the SMP. The median tongue dose was significantly reduced (29.6 Gy vs. 34.3 Gy). The volumes of the tongue receiving a dose of 15 Gy, 30 Gy, 35 Gy, 45 Gy, and 60 Gy were significantly lower than using the SMP.

**Conclusion:**

The SCTDD significantly decreased the radiation dose to the tongue compared to the SMP, which may potentially reduce RT-related tongue toxicity.

## Introduction

Radiation therapy (RT) has played an important role as a standard treatment for head and neck squamous cell carcinoma (HNSCC) [1–4] with surgery and chemotherapy. However, it is not an easy task to meet the aim of RT, which delivers a curable dose to a target volume while minimizing the dose to organs at risk (OARs) near the target volume because head and neck (H&N) tumors usually overlap or are adjacent to normal organs. There are many OARs of concern in the RT planning for H&N cancer, such as the brain, brainstem, optic apparatus, parotid gland, submandibular glands, pharyngeal muscles, laryngeal structures, and oral cavity (OC), including the tongue. Intensity modulated radiation therapy (IMRT) technique has made a significant contribution to reduce dose of these OARs [1, 2, 5–7], but reduction of OC dose to a meaningful level still remain a big challenge because it is close to target volume and immobilization is not an easy task in H&N RT.

The tongue is a subsite of the OC and is not separately described in the OAR delineation guidelines [8]. However, it plays important roles in taste, saliva production, speech, and swallowing [5, 7, 9, 10], so it is important to minimize the radiation dose to the tongue for better quality of life following RT. High mean dose of the tongue is closely related to movement of tongue and taste dysfunction that affect quality of speech and weight loss respectively in H&N RT [9, 10]. Percentage relative taste loss was not observed until radiation doses of 20 Gy had been reached. Between 20 Gy and 40 Gy, taste loss increased rapidly, while over 90% relative taste loss was observed at the 60 Gy dose level [11, 12]. Shi. et al. reported that significant impaired threshold of taste was revealed at 30 Gy [12].

Some clinical strategies have been used to reduce the tongue dose in H&N RT. First, radiation dose optimization permits protection of the tongue by employing advanced RT techniques, such as intensity-modulated radiation therapy (IMRT) [6, 7]. However, when the tongue is adjacent to the target volume, H&N RT can be challenging. Second, Kil et al. reported that the ‘stick-out’ position of the tongue without an intraoral device (IOD) can be useful to reduce the tongue dose, especially for tongue base dose reduction [13]. However, it does not seem likely that good position reproducibility will be provided during IMRT with effective tongue displacement to the contralateral side of the target. Finally, as an active approach, a technique that can intentionally displace the tongue from the target volume can be used by employing a tongue displacement device (TDD) during RT [14].

**Fig. 1:**
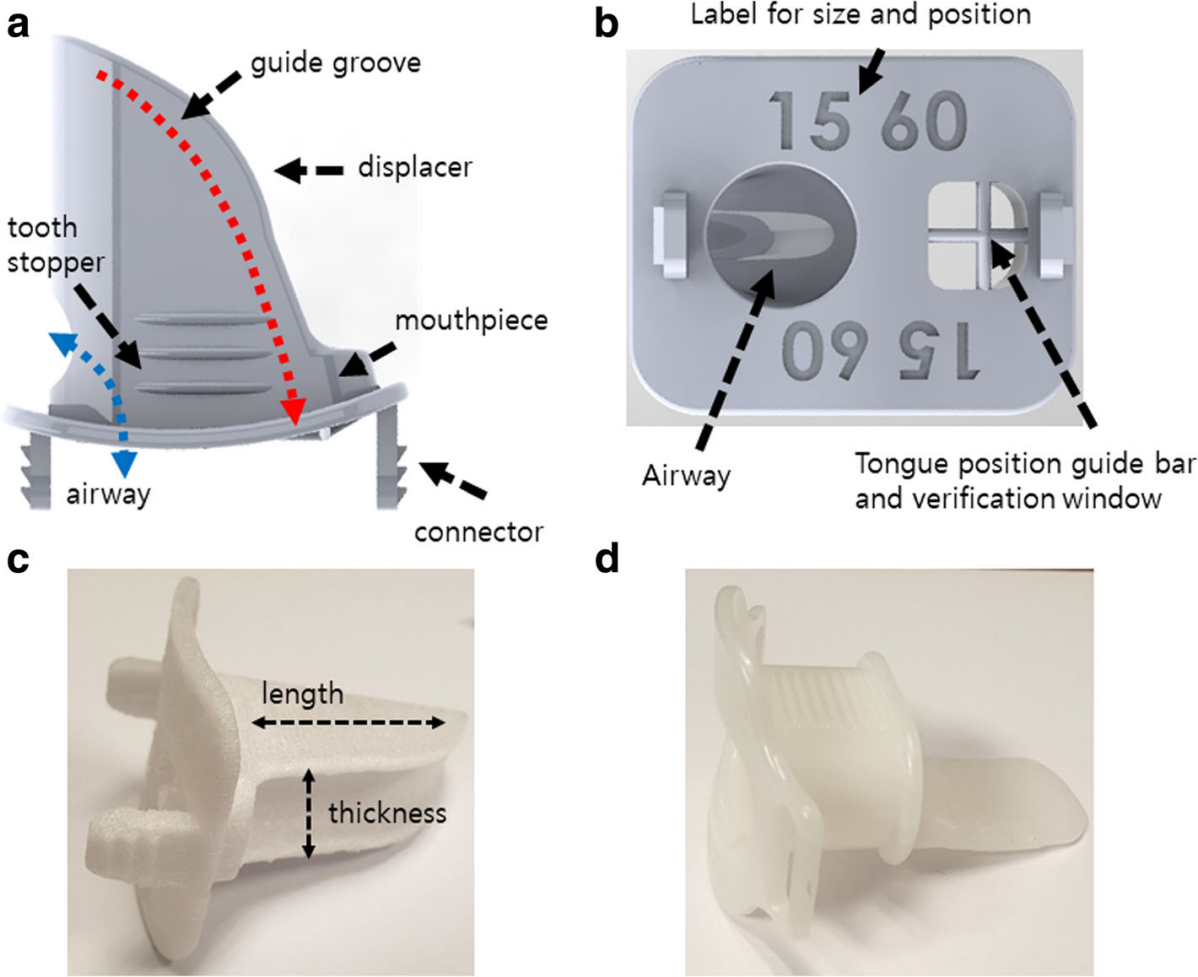
**a** Top and **b** front views of the 3D model for the semi-customized tongue displacement device. It was printed using a 3D printer with a biocompatible material (**c**). **d** Commercially available standard mouthpiece, which has been the most commonly used device in H&N RT

Traditionally, a standard IOD (SIOD), such as a mouthpiece, bite-block, or tongue blade, has been used to reduce the dose and enhance immobilization of the tongue by displacement and/or compression of the tongue [14–19]. However, these have some limitations for clinical implementation. First, a commercially available standard mouthpiece (SMP, Fig. 1d), which has been the most commonly used SIOD, is not effective to displace and/or immobilize the tongue during HNSCC RT because it is designed for different purposes, such as an endoscopy mouthpiece or a tooth protector. Displacement of the tongue to the contralateral side of the planning target is very effective to reduce the dose to the tongue in ipsilateral H&N irradiation for well-lateralized H&N cancer, such as tonsil and OC cancer, but it is impossible to meet this goal with a commercially available SIOD. Finally, an SIOD is not suitable for patient-specific customization. Patients have different OC structures, and jaw opening depends on tumor location and size and surgery conditions. If the IOD does not fit well with the OC structure and its purposes, it does not provide patient comfort or dosimetric benefit. Therefore, to solve these problems, a patient-specific TDD is needed. Janson and Bodard reported that dosimetric benefits with patient comfort were obtained by using patient-specific IODs supplied by dental services for H&N RT [14, 16]. However, it is not an easy task for clinical implementation because it requires a long waiting time with high cost. In addition, such dental service is not available for all RT facilities.

In order to displace and immobilize the tongue in ipsilateral H&N RT for well-lateralized HNSCC, we developed a unique TDD that can be semi-customized for various patient conditions using 3D printing technology. The manufacturing process of the semi-customized TDD (SCTDD) was introduced, and dosimetric characteristics were compared with those of an SMP.

## Materials and methods

### Development of a patient-specific semi-customized tongue displacement device

We designed a unique SCTDD combining the merits obtained from the many papers mentioned in the introduction and our clinical experience. The major concerns of the SCTDD design were displacement and sticking out, simple position verification, and robust immobilization of the tongue with patient-specific semi-customization for patient comfort. For these, the SCTDD consists of three parts: a mouthpiece, connector, and tongue displacer (Fig. 1). The mouthpiece has tooth stoppers on the upper and lower sides of the mouthpiece, an airway, and a position guide bar with a position verification window for the tongue. A tooth stopper, which is the contact point of the patient’s teeth, was designed to improve reproducibility of the biting position and prevent teeth from slipping. When the patient bites the tooth stopper using central incisors and then sticks out the tongue until the tip reaches the guide bar, the tongue moves along the guide groove of the displacer (red dotted arrows in Fig. 1). As a result of this, the tongue is naturally displaced to the contralateral side of the target. In order to improve the interfractional reproducibility of the tongue position, a verification window was designed to verify the position of the tip of the tongue during patient setup. To ensure natural breathing of the patient during treatment, we added an airway on the front of the mouthpiece, starting from the opposite side of the SCTDD displacer. Finally, we designed two connectors that can be attached to the immobilization mask on the front panel of the mouthpiece.

For patient-specific semi-customization of the TDD, different sizes of the SCTDD were designed by combining different thicknesses from 5 mm to 20 mm in 5 mm increments and lengths from 40 mm to 70 mm in 10 mm increments to cover different jaw openings and lengths of the OC (Fig. 1c), respectively. For simplification of the manufacturing process, the SCTDD was designed using a computer-aided design (CAD) program and converted into a stereolithography file format, which can be identified by a three-dimensional printer (3DP). The designed SCTDD was printed using a fused deposition modeling 3DP (3DISON Multi, Rokit, Korea) with a biocompatible 3DP material (Kitchen & Deco, Rokit, Korea).

We measured the average 3D printing times, excluding the modeling, to evaluate the efficiency of the SCTDD manufacturing procedure for clinical implementation.

### Patient selection and simulation

This dosimetric study did not involve any human or animal experiments. With approval from the institutional review board, between June 2016 and October 2016, seven consecutive patients, three for tonsil cancer and four for OC cancer with histologically proven HNSCC, who underwent ipsilateral H&N RT with helical tomotherapy (TomoHD™, Accuray, USA) were included in this study (Table 1). All methods were performed in accordance with the institutional review board for retrospective study. All of this process have been performed on the informed consent. All patients were immobilized by an individually customized thermoplastic mask (Aquaplast RT™, Q-fix, USA) with SCTDD and SMP in the supine position.

**Table 1:** Patients’ characteristics

Case	Gender	Age	Primary Site	Clinical Stage	Histology	Aim of RT
1	Male	40	Tonsil	cT2N2bM0	Squamous	Definitive
2	Female	46	Oral cavity	pT4aN0M0	Squamous	Adjuvant
3	Male	56	Tonsil	cT1N2aM0	Squamous	Definitive
4	Male	64	Tonsil	cT2N1M0	Squamous	Definitive
5	Male	54	Oral cavity	pT4aN2bM0	Squamous	Adjuvant
6	Female	60	Oral cavity	pT2N1M0	Squamous	Adjuvant
7	Male	72	Oral cavity	pT2N1M0	Squamous	Adjuvant

*Abbreviation*: *RT* radiation therapy

An SCTDD was fitted to the patient by an internal guideline as described in the following procedures. Before the planning computed tomography (CT), a SCTDD was selected based on the physical length of the jaw opening (upper to lower incisor) and depth of the OC in the diagnostic CT, which correspond to the thickness and length of the SCTDD, respectively.

For correct use and elimination of the sense of rejection by the SCTDD, the patient was trained using their own SCTDD for 10 min according to the design purposes described in the previous session. After the patient was familiarized with the SCTDD, the tongue was stuck out as much as possible until the tip reached the tongue position guide bar. After confirming this through the tongue position verification window, the patient was immobilized using a thermoplastic mask in conjunction with the connector of the SCTDD to improve the interfractional reproducibility.

For the SMP, most procedures were the same as for the SCTDD, but a single sized standard model was used. In addition, the SMP was designed to depress the tongue and immobilize it without position verification (Fig. 1d). Two sets of planning CT images were obtained under the same scan conditions with an SCTDD and an SMP for each patient and were transferred into TPS (Pinnacle3®, version 9.2; Philips Medical System, USA) for contouring.

### Treatment planning

For tonsil cancer patient-aimed definitive RT, the gross tumor volume (GTV) and clinical target volume (CTV) were delineated on both CT image sets, while only CTV was delineated for OC cancer patient-aimed adjuvant RT. The planning target volumes (PTV, P_GTV and P_CTV) were generated with an isotropic expansion of 3 mm from GTV and CTV, respectively, which were modified so that the expanded PTVs did not exceed the actual anatomic boundaries, such as the spinal cord and skin surface. OAR, including the spinal cord, brainstem, parotid gland, and tongue, were delineated on both CT image sets according to internal guidelines based on a reference [8]. The planning volume for the spinal cord (P-cord) was generated by adding a 3-mm margin to the actual spinal cord. All contours were delineated by a radiation oncologist based on the same rules for consistency.

For dose planning, all contour data with the planning CT was transferred to TPS for tomotherapy (Tomotherapy, Accuray, USA). For dosimetric comparison, helical tomotherapy plans were generated for both the SCTDD and SMP with a 6 MV photon beam. The same dose constraints and prescriptions were used in both plans based on internal guidelines as follows: total doses of 66 Gy and 60 Gy were prescribed to P_GTV and P_CTV, respectively, in 33 fractions using the simultaneously integrated boost technique for tonsil cancer, while a total dose of 59.4 Gy was prescribed to P_CTV in 27 fractions for OC cancer.

Two constraints were set at the highest priority level: 95% of PTVs should receive at least 100% of the prescription dose, and the maximum dose to P-cord should not exceed 45 Gy (D_max_ < 45 Gy). In order to achieve the most homogenous dose distribution as possible within and around the PTV, 99% of the PTV volume should receive at least 95% of the prescription dose, and the volume receiving ≥110% of the prescribed dose should not be greater than 1 cm^3^ in total volume. The constraints at the second priority level were to limit the mean dose (D_mean_) under 26 Gy and 30 Gy for the parotid and tongue, respectively. The lowest level constraint was to limit the dose to the brainstem: D_max_ should not exceed 54 Gy.

For each plan, the same number of iterations was used during the dose optimization process. During inverse planning, once PTV constraints were reached, the optimization was continued to reduce the doses to OAR until the iteration limit was reached while maintaining the PTV dose. In both plans, a field width of 2.5 cm, modulation factor of 2.0, and pitch of 0.287 were used to avoid the thread effect [20]. Dose calculation was conducted using the collapsed-cone convolution algorithm [21, 22].

### Evaluation of the geometric and dosimetric effects of the SCTDD

We evaluated the geometric and dosimetric effects of the SCTDD compared with those of the SMP. For comparative evaluation of the geometrical change on the tongue, tongue volume and percent of tongue volume outside the PTV were measured in both the SCTDD and SMP plans.

In order to evaluate the effect of the SCTDD on plan quality, the minimum dose received by 95% (D_95_), 50% (D_50_), and 2% (D_2_) of the PTVs in the SCTDD plan were compared with those of the SMP plan data. The homogeneity index (HI = (D_2_-D_98_)/D_50_) was also compared. In addition, several relevant dosimetric parameters on these OAR were used for comparison: D_mean_ of the parotid; D_max_ of the P-cord and brainstem; and the percentages of tongue volume that received a dose of 15 Gy (V_15_), 30 Gy (V_30_), 35 Gy (V_35_), 45 Gy (V_45_), 60 Gy (V_60_), or more; and D_max_ and D_mean_ of the tongue.

Statistical analysis of the dosimetric comparison between the SMP and SCTDD was done using a Wilcoxon signed-rank test. A probability level with a *p*-value < 0.05 was considered significant.

## Results

### Geometrical effect of the semi-customized tongue displacement device

We designed (Fig. 1a and b) and printed (Fig. 1c) 16 SCTDD models with different depths and lengths to cover various patient conditions. 3D printing of the SCTDD took a minimum of 60 min and a maximum of 120 min depending on the SCTDD size. Additionally, approximately 30 min of post processing were required.

**Fig. 2:**
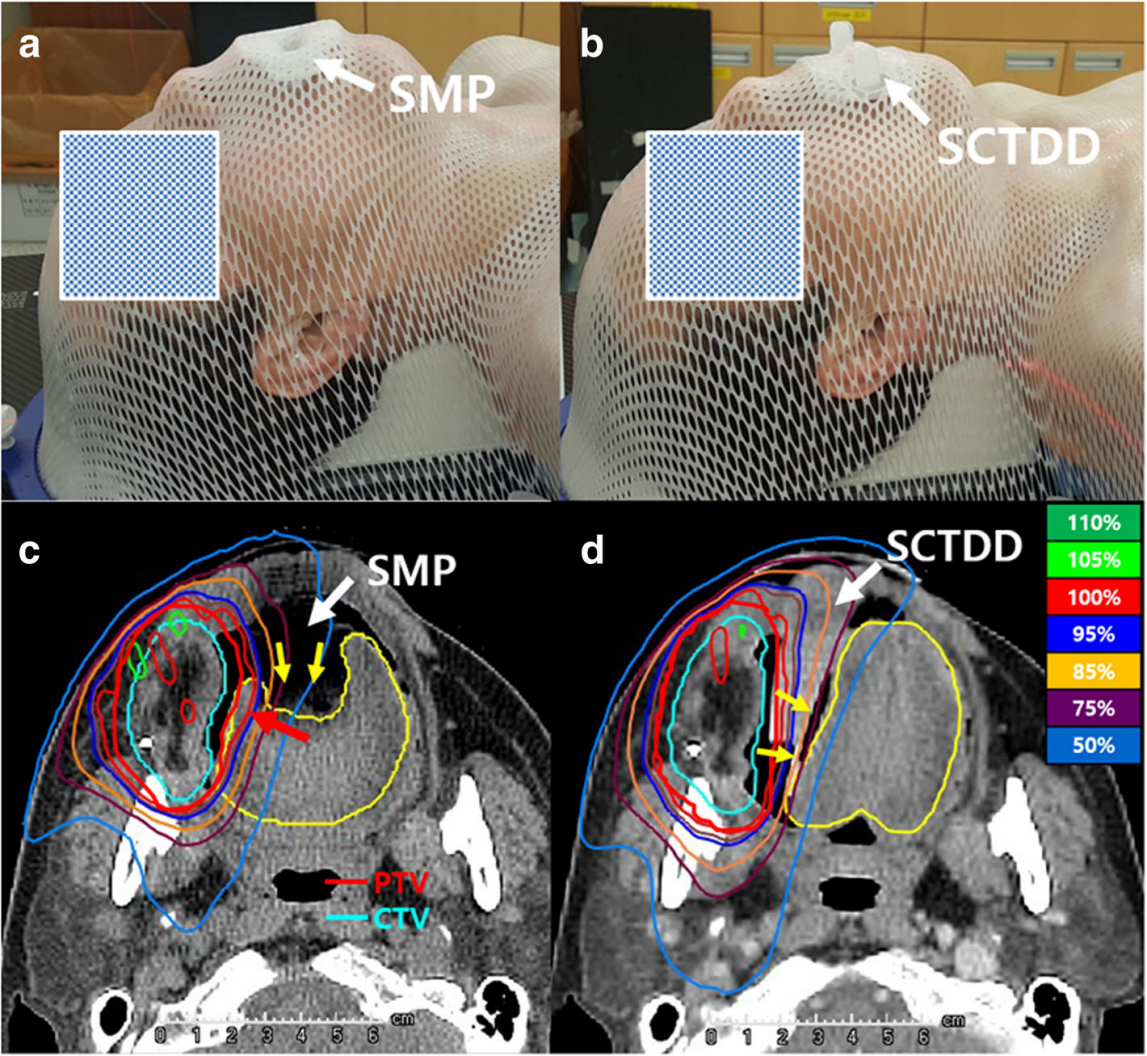
Patient setup photograph with **a** a standard mouthpiece (SMP) and **b** semi-customized tongue displacement device (SCTDD) for a patient who underwent H&N tomotherapy. Isodose distributions of this patient in the axial section for the **c** SMP and **d** SCTDD plans are also shown. The tongue was effectively displaced from the high-dose region near the planning target volume (yellow arrows) by using the SCTDD, while a partial volume of the tongue received a high dose equivalent to the prescribed dose (red arrow) in the SMP plan because SMP only depressed tongue (yellow arrow) without displacing it to the contralateral side of the target

The average thickness and length of the SCTDD for seven patients were 2.1 cm and 6.8 cm, respectively. The connector of the SCTDD (Fig. 2b) provided better fixation with a thermoplastic mask compared with the SMP (Fig. 2a). Using an SMP, depression and immobilization of the tongue were possible in all patients, but displacement of the tongue from PTV (planning target volume) was not effective (Fig. 2c). In contrast, using an SCTDD, the tongue was effectively displaced from PTV and immobilized in all patients (Fig. 2d). These effects were well represented in the statistical analysis related to tongue volume as follows. The median tongue volumes were similar between the SMP and the SCTDD (82.6 cm^3^ vs. 80.5 cm^3^, *p* = 0.578). However, a significant increase in median percentage of tongue volume outside the PTV was observed in the SCTDD compared with the SMP (91.3% vs. 86.4%, *p* = 0.016) (Table 2).

**Table 2:** Comparison of geometric and dosimetric characteristics for tongue: SMP versus SCTDD

Parameters	SMP	SCTDD	*p*-value
Tongue volume	82.6 cm^3^ (73.1, 95.4)	80.5 cm^3^ (77.7, 100.5)	0.578
Percent of tongue volume outside the PTV	86.4% (84.9, 90.8)	91.3% (87.3, 91.9)	0.016^a^
V_15_	89.7% (83.3, 94.3)	79.9% (71.7, 81.9)	0.016^a^
V_30_	48.3% (46.1, 53.3)	37.8% (34.1, 40.9)	0.016^a^
V_35_	41.4% (37.9, 47.1)	30.6% (27.2, 34.5)	0.016^a^
V_45_	29.4% (27.4, 36.6)	22.7% (17.2, 24.2)	0.016^a^
V_60_	13.7% (8.0, 16.1)	7.4% (6.6, 12.6)	0.016^a^
D_mean_	34.3 Gy (33.0, 35.5)	29.6 Gy (27.3, 30.4)	0.016^a^
D_max_	62.9 Gy (62.3, 69.3)	62.3 Gy (62.1, 69.0)	0.078

*Abbreviations: IQR* interquartile range, *SMP* standard Mouthpiece, *SCTDD* semi-customized tongue displacement device, *V*_*D (15, 30, 35, 45, and 60)*_ the percentage of the tongue volume that received D (15, 30, 35, 45, and 60) Gy or more, *D*_*mean*_ mean dose, *D*_*max*_ maximum dose.

^a^Statistically significant

Values are presented as median (IQR Q1, Q3)

### Dosimetric comparison between the SMP and SCTDD

By comparing the SCTDD with the SMP, the dosimetric characteristics for the PTVs, parotid grands, P-cord, and brainstem were calculated, as summarized in Table 3. For target dose coverage, no significant dose differences between the SCTDD and SMP were observed in D_98_, D_50_, and D_2_ for all PTVs (*p* > 0.05). In addition, no significant dose difference was observed in median of D_mean_ for the parotid (15.5 Gy vs. 15.3 Gy, *p* = 0.578) or median of D_max_ for P-cord (27.1 Gy vs. 26.6 Gy, *p* = 0.938) and the brainstem (21.3 Gy vs. 19.2 Gy, *p* = 0.469).

**Table 3:** Comparisons of dosimetric characteristics

Parameters			SMP	SCTDD	*p*-value
Tonsil Cancer	P_GTV	D_2%_ (Gy)	68.5 (68.3, 68.6)	68.3 (68.0, 68.4)	0.205
		D_98%_ (Gy)	65.7 (65.6, 65.7)	65.7 (65.6, 65.7)	0.750
		D_50%_ (Gy)	67.3 (67.2, 67.3)	67.1 (67.1, 67.2)	0.250
		HI	0.042 (0.039, 0.043)	0.039 (0.034, 0.042)	0.250
	P_CTV	D_2%_ (Gy)	68.1 (68.0, 68.1)	67.9 (67.7, 67.9)	0.250
		D_98%_ (Gy)	59.5 (58.8, 59.8)	58.5 (58.4, 59.2)	0.250
		D_50%_ (Gy)	63.5 (63.3, 63.8)	63.0 (62.5, 63.6)	0.250
		HI	0.136 (0.131, 0.144)	0.149 (0.136, 0.152)	0.250
Oral Cavity cancer	P_CTV	D_2%_ (Gy)	62.2 (61.8, 62.3)	62.0 (61.7, 62.2)	0.125
		D_98%_ (Gy)	58.7 (58.6, 58.8)	58.7 (58.7, 58.8)	0.875
		D_50%_ (Gy)	60.9 (60.6, 61.0)	60.8 (60.6, 61.0)	0.375
		HI	0.058 (0.050, 0.060)	0.054 (0.048, 0.058)	0.625
Parotid glands		D_mean_ (Gy)	15.3 (13.4, 15.7)	15.5 (13.1, 16.9)	0.578
P-cord		D_max_ (Gy)	26.6 (24.7, 30.7)	27.1 (23.4, 30.5)	0.938
Brainstem		D_max_ (Gy)	19.1 (15.0, 22.3)	21.3 (15.5, 23.4)	0.469

Values are presented as median (IQR Q1, Q3)

In connection with geometrical change of the tongue by using an SCTDD, the tongue was effectively displaced from the high dose region near PTV (Fig. 2d), while a partial volume of the tongue received a high dose similar to the prescribed dose in the SMP (Fig. 2c). The median of D_mean_ for the tongue was significantly reduced by 15.9% in the SCTDD (29.6 Gy, IQR: 27.3, 30.4) compared with the SMP (34.3 Gy, IQR: 33.0, 35.5) (*p* = 0.016) (Table 2). Moreover, median of V_15_, V_30_, V_35_, V_45_, and V_60_ for the tongue were significantly lower in the SCTDD (79.9, 37.8, 30.6, 22.7, and 7.4%, respectively) than the SMP (89.7, 48.3, 41.4, 29.4, and 13.7%, respectively) (all *p* < 0.05). However, no significant difference in median of D_max_ for the tongue was observed between the SCTDD (62.3, IQR: 62.1, 69.0 Gy) and the SMP (62.9, IQR: 62.3, 69.3) (*p* = 0.078).

## Discussion

The tongue plays a very important role in taste, speech, and swallowing functions. Reducing the radiation dose to the tongue can retain these functions following RT. Several authors have shown evidence of this. Sapir et al. reported significant association between dysgeusia and radiation dose to the tongue [9]. Jacobi et al. showed that changes in speech are related to mean doses to the tongue [10]. Schwartz et al. suggested that V_30_ < 65% and V_35_ < 35% for the anterior OC are predictive factors for swallowing dysfunctions [23]. Eisbruch et al. showed that the mean dose to the OC, representing the RT effect on the minor salivary glands, is a significant factor of dry mouth [5]. In the current study, the use of the SCTDD decreased the mean dose to the tongue through V_15_ to V_60_ compared to the SMP without sacrifice of plan quality or patient setup stability.

IOD has been playing an important role in reducing the dose of oral cavity and its subsites, as well as improving setup uncertainty [24, 25]. For effective working of the IOD, it should cover various patient conditions such as different jaw opening and complex oral cavity structure based on surgery result. So, we developed unique SCTDD manufacturing process that can be realized in clinic based on semi-customization using a CAD software and 3D printing technology. For semi-customization, we first designed an ideal model of the SCTDD, and then created many different size SCTDD models to fit various patient conditions using copy, resizing, and modification tool provided by commercial CAD software. Furthermore 3D printing technology made it possible to manufacture these models in short time with low cost by clinical staff, that were not possible in conventional manufacturing process such as milling or casting process. Our proposal is useful to handle unexpected patient condition because SCTDD model can be modified and printed within half day if prepared SCTDD does not fit the patient condition.

There are some considerations for clinical implementation of the SCTDD. For accurate customization of the TDD, the most important factor is to select the proper thickness and length of the SCTDD for a patient. Until now, we applied an SCTDD to 17 patients; seven in this study and 10 not included to whom only SCTDD were applied so that comparison with SMP could not be performed. In these 17 patents, 12 of 13 patients for definitive setting had SCTDDs with thicknesses of 2~2.5 cm and lengths of 6.5~ 7 cm, while one patient had an SCTDD with a thickness of 1.5 cm and length of 6.5 cm. Of four patients for post-op setting, three had an SCTDD with a thickness of 1 cm and a length of 6.5 cm, while one had an SCTDD with a thickness of 1.5 cm and a length of 7 cm. In patients for post-op setting, it was not possible to apply an SMP because they had difficulty opening the jaw sufficiently, while an SCTDD was very easy to apply. In this context, we can categorize the SCTDD size into two groups: one with a thickness of 2.25 cm and a length of 6.75 cm, which is very close to results of seven present patients, for definitive RT patients and one with a thickness of 1.25 cm and a length of 6.75 cm for post-op patients.

The SCTDD is placed with a patient’s mouth open. As the course of RT progresses, oral discomfort or pain may increase because of oral mucositis and trismus caused by RT. For this reason, the SCTDD should have a rounded edge design with a smooth surface. Additionally, biocompatible and soft material is recommended for 3D printing, with smooth surface treatment as a post processing step. Fortunately, various types of 3D printing material that meet these requirements are commercially available, and this will be broaden by rapid advancement of 3D printing technology.

Position reproducibility of the tongue with the SCTDD is also very important, although it is not included in this study. To obtain robust positional reproducibility, we introduced two key points into the design of the SCTDD: the connector, which connects to a thermoplastic mask, and a tongue position guide bar with a verification window. Both of these features were very effective at maintaining positional reproducibility of the SCTDD and the tongue, as well as reduction of patient setup time. Evaluation of the long-term positional reproducibility can be contributed to improve the SCTDD design based on the setup image.

## Conclusions

By employing 3D printing technology with an in-house manufacturing process, a unique SCTDD was developed to displace and immobilize the tongue for ipsilateral H&N RT. It significantly decreased the radiation dose to the tongue compared to an SMP and could potentially reduce RT-related tongue toxicity. Furthermore, with rapid advancement of 3D printing technology, this investigation may also contribute to improve manufacturing processes for patient-specific customized devices for RT.
